# Improving *In Vitro* Digestibility and Reducing Immunogenic Peptide Exposure in Soybean Meal through Exogenous Protease Supplementation

**DOI:** 10.1021/acs.jafc.6c05174

**Published:** 2026-06-29

**Authors:** Leila Zafra, Cristina Jiménez-Holgado, Bindhu Lakshmibai Vasanthakumari, Beatriz Miralles, Marta Martinez-Sanz, Isidra Recio

**Affiliations:** † Instituteof Food Science Research, CIAL (CSIC-UAM, CEI UAM+CSIC), C. Nicolás Cabrera, 9, 28049 Madrid, Spain; ‡ Escuela de Doctorado, Universidad Autónoma de Madrid (UAM), C. Francisco Tomas y Valiente, 7, 28049 Madrid, Spain; § 57852 Kemin Industries, 1900 Scott Ave, Des Moines, lowa 50317, United States

**Keywords:** soybean meal, protein digestibility, proteases, allergens, *in vitro* digestion, peptidomics

## Abstract

Exogenous proteases can enhance protein digestion in feed. However, their precise impact on peptide release and allergenecity remains poorly understood. This study evaluated the effects of a protease blend (Protease I) and a single protease (Protease II) on soybean meal *in vitro* digestibility and peptide release, using the standardized INFOGEST model. Compared to the Control, Proteases I and II increased protein digestibility from 83–89% to 90–96% and 91–92%, respectively, while enhancing peptide release from typically enzyme-resistant regions. Peptide profiling showed broader sequence coverage of key soybean proteins and revealed distinct substrate specificities. Cleavage site analysis indicated that exogenous enzymes altered the proteolytic pattern. Importantly, epitope sequence comparison revealed that enzymatic hydrolysis reduced the presence of certain allergenic epitopes. These findings suggest that protease supplementation enhances digestibility and may reduce allergenic potential, offering a promising strategy to improve the nutritional quality and safety of plant-based feed proteins.

## Graphical abstract

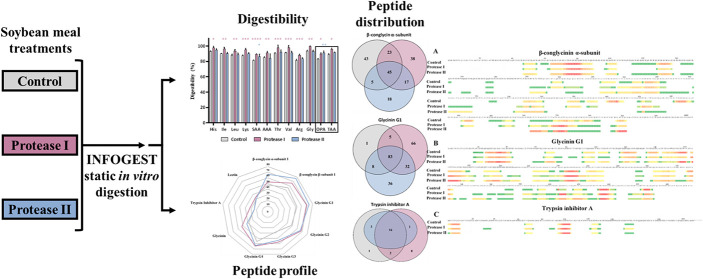

## 1 Introduction

The assessment of protein quality, particularly in terms of digestibility, is crucial for maintaining and enhancing overall health and well-being.^1^ As the demand for sustainable protein sources grows, plant-based proteins, and especially those derived from legumes, are gaining prominence.^2,3^ Among these, soybean is one of the most widely used sources, owing to its high protein content, favorable amino acid (AA) profile, and cost-effectiveness.^4^ Soybean is not only increasingly recognized in the human diet^5^ but also plays a key role in animal nutrition, serving as the primary protein source in nonruminant feed formulations worldwide, particularly in swine and poultry diets.^6–8^ Soybean meal, the main byproduct of oil extraction, typically contains 44–48% crude protein and is the most commonly used protein-rich feed ingredient in monogastric nutrition.^9^ However, despite its nutritional advantages, the full nutritional potential of soy protein is limited by the presence of several antinutritional factors (ANFs), including trypsin inhibitors, lectins, glycinin, β-conglycinin, oligosaccharides, phytoestrogens, and phytates.^10,11^ These compounds can impair protein digestion and absorption, disrupt nutrient metabolism, reduce growth performance, and even trigger adverse physiological reactions, thereby limiting its application potential.^12–14^ In particular, glycinin and β-conglycinin—the main storage globulins in soybean^15^—are partly resistant to enzymatic digestion due to their structural stability conferred by disulfide bonds.^16^ These proteins are also known allergens that can provoke immune responses and modify intestinal morphology, further compromising nutrient utilization and animal performance.^17–19^ To mitigate these limitations, various processing and supplementation strategies have been proposed to reduce the activity of ANFs and improve the digestibility of soy protein. Traditional approaches include heat treatment, which can inactivate some ANFs such as trypsin inhibitors.^20^ However, excessive heating may indiscriminately destroy other essential nutrients present in the legume and can lead to the degradation of heat-sensitive AA if not carefully controlled, thereby compromising their nutritional value.^21–25^ Fermentation and enzymatic hydrolysis have also been explored as viable alternatives, with enzymatic supplementation—particularly the use of exogenous proteases—gaining interest as an effective and widely adopted strategy to enhance protein digestibility and utilization, as well as to reduce feed costs in livestock production, and potentially reduce the presence of immunogenic peptides while preserving nutritional quality.^26–31^ Proteases facilitate the breakdown of complex proteins into absorbable peptides and AA, thereby improving nutrient bioavailability, promoting gut health, and supporting growth performance.^29,30,32^ Specifically, in broiler chickens, dietary protease supplementation has been associated with better AA utilization and enhanced intestinal morphology.^33–35^ Despite growing interest and promising *in vivo* results, standardized *in vitro* digestion models are necessary to evaluate protease efficacy more systematically and to understand their interactions with specific soybean proteins. Furthermore, while the general benefits of proteases on animal performance are well-known,^36,37^ the specific activities on enzyme-resistant regions and their effects on individual allergenic epitopes is still unexplored under standardized physiological conditions. Thus, investigating these mechanisms through advanced peptidomic profiling and epitope mapping provides novel insights into which sequences are released or degraded, offering a deeper understanding of protein quality and potential allergenicity. To address this gap, the primary objective of the current study was to comprehensively evaluate and compare the efficacy of two different enzymatic strategies on soybean meal using the standardized INFOGEST gastrointestinal simulation model. Specifically, we compared a multiprotease blend containing acidic, neutral, and alkaline enzymes derived from *Aspergillus niger*, *Bacillus subtilis*, and *Bacillus licheniformis* (Protease I) against a single alkaline protease derived solely from *B. licheniformis* (Protease II). This direct comparison holds practical significance for the feed industry, as it contrasts a multienzyme complex, designed to target a broader range of peptide bonds, with a specialized, single-source protease. Accordingly, this study aimed to determine their distinct impacts on *in vitro* protein digestibility, cleavage patterns, and the potential exposure or reduction of immunogenic peptide fragments.

## 2 Materials and Methods

### 2.1 Materials

A soybean meal (protein content of 46.1%, determined by Kjeldahl) and a set of two proteases (Protease I, a multiprotease blend consisting of acidic, neutral and alkaline proteases produced from *A. niger*, *B. subtilis* and *B. licheniformis* respectively, and Protease II, an alkaline protease derived from *B. licheniformis*) were provided by Kemin Industries (Des Moines, Iowa, USA). For electrophoretic analysis, Precision Plus Protein Unstained Standards, 12% Bis-Tris polyacrylamide gels, and XT-MES running buffer were obtained from Bio-Rad Laboratories (Richmond, CA, USA), while Coomassie Blue (Instant Blue) was sourced from Expedeon Protein Solutions (Harston, UK), and the loading buffer was prepared with reagents sourced from Merck (Darmstadt, Germany). Pepsin from porcine gastric mucosa, pancreatin from porcine pancreas, and porcine bile extract, as well as standard reagents for acid hydrolysis and AA analysis, were purchased from Sigma-Aldrich (St. Louis, MO, USA), whereas derivatization was performed using the AccQ-Tag Ultra reagent in phosphate buffer (Waters Corporation, Milford, MA, USA). Trifluoroacetic acid (TFA), formic acid, and trichloroacetic acid (TCA) were purchased from Thermo Fisher Scientific (San Jose, CA, USA), and LCMS-grade water, methanol, and acetonitrile were acquired from VWR International (Radnor, Pennsylvania, USA). Hydrolysis-compatible glass vials and 100 μL bed volume ZipTipC18 pipet tips were sourced from Agilent Technologies (Santa Clara, CA, USA), and 0.45 μm filters were obtained from Branchia (Barcelona, Spain). Analytical equipment included an ES-20/60 orbital shaker-incubator (Biosan, Riga, Latvia), a 5804R refrigerated centrifuge and a thermoblock (Eppendorf, Hamburg, Germany), a Savant SpeedVac SPD120 vacuum concentrator (Thermo Fisher Scientific, Asheville, NC, USA), and a forced air-drying oven (LBX Instruments, Barcelona, Spain). Final analytical quantifications were performed using a VANTAstar microplate reader (BMG LABTECH, Ortenberg, Germany) and a Vanquish UHPLC system equipped with a UV detector (Thermo Fisher Scientific, Reinach, Switzerland) equipped with an AccQ-Tag Ultra analytical column (2.1 mm × 100 mm, 1.7 μm, Waters Corporation, Milford, MA, USA), and peptidomic profiling was carried out on a Vanquish Neo nano-UHPLC system coupled to an Orbitrap Exploris 240 mass spectrometer (Thermo Fisher Scientific, San Jose, CA, USA) using an Easy-Spray PepMap Neo C18 column (50 cm length, 2 μm i.d. × 75 μm, Thermo Fisher Scientific, San Jose, CA, USA). No unexpected significant hazards were associated with the experimental work.

### 2.2 Analysis of Total Nitrogen by Kjeldahl

Total nitrogen in soybean meal was conducted by the Kjeldahl method according to the ISO norm.^38^ Protein content was subsequently calculated using a nitrogen-to-protein conversion factor of 6.25.^39^

### 2.3 Sodium Dodecyl Sulfate–Polyacrylamide Gel Electrophoresis (SDS-PAGE)

SDS-PAGE analysis was performed according to the method described in ref 40. In summary, products and ingredients were dissolved in a loading buffer composed of 0.05 M Tris-HCl (pH 6.8), 1.6% (w/v) SDS, 8% (v/v) glycerol, 2% (v/v) β-mercaptoethanol, and 0.002% (w/v) bromophenol blue, adjusted to a final protein concentration of 0.8 mg/mL. Samples were then heated to 95 °C for 5 min in a thermoblock. After centrifugation at maximum speed, 40 μL of each sample and 15 μL of molecular weight marker were loaded onto a precast 12% Bis-Tris polyacrylamide gel and electrophoresed at 150 V for 45 min using XT-MES 20× as the running buffer. The gel was rinsed with Milli-Q water, stained with Coomassie Blue for 1 h, and then left to soak overnight.

### 2.4 *In Vitro* Simulated Gastrointestinal Digestions

A nontreated (Control) and two protease-treated (Protease I and Protease II) soybean meal samples were prepared by adding the solid enzymes to the meal suspension at a dose of 30 g/kg. Then, samples were subjected to *in vitro* gastrointestinal digestions in triplicate according to the INFOGEST protocol,^41^ with specific modifications. Specifically, digestion times were adjusted to 1 h for the gastric phase and 3 h for the intestinal phase (instead of the standard 2 h/2 h) to better reflect the avian digestive transit.

However, pH and enzyme activities were kept under standard INFOGEST conditions to ensure reproducibility and avoid multivariable kinetic complexity, and while using commercial porcine enzymes is an acknowledged limitation due to distinct avian kinetics, this approach provides a robust baseline for comparative screenings. In addition, the amount of sample was adjusted to achieve a 4% protein concentration in the oral phase, doubling the standard 2% concentration typically recommended by the protocol. This higher substrate load was selected based on recent findings in digestibility studies of protein isolates containing trypsin inhibitors to better reflect physiological digestion,^42^ which have demonstrated that in samples containing trypsin inhibitors, increasing the protein input can affect trypsin activity without compromising overall digestibility, thereby allowing for a more robust evaluation of inhibitor-rich plant ingredients. To simulate this phase, 1 mL of simulated salivary fluid (SSF, pH 7) and 1 mL of substrate suspension in Milli-Q water were added, and the mixture was incubated at 37 °C for 2 min in an orbital shaker-incubator set to 130 rpm. Subsequently, 1.6 mL of simulated gastric fluid (SGF, pH 3) was introduced, the pH was adjusted to 3, and 1 μL of calcium chloride, pepsin from porcine gastric mucosa (final pepsin activity 2000 U/mL of gastric digest), and enough Milli-Q water to reach a final volume of 4 mL were added. The samples were then incubated for 1 h under the previously described conditions. Gastric digestion was halted by raising the pH to 7 using 1 M NaOH. Following this, 1.7 mL of simulated intestinal fluid (SIF, pH 7) was added, the pH was adjusted to 7, and pancreatin from porcine pancreas (final trypsin activity 100 U/mL of intestinal digest) and porcine bile extract (final bile salts concentration 10 mM of intestinal digest) were incorporated along with 8 μL of calcium chloride to initiate the intestinal phase. The total volume was adjusted to 8 mL with Milli-Q water, and the samples were incubated for an additional 3 h under the same conditions. Next, 32 mL of pure methanol were added, and after a 1 h incubation at −20 °C, the samples were centrifuged at 4000*g* for 15 min at 4 °C, yielding two fractions: the nonabsorbable fraction (pellet) and the absorbable fraction (supernatant). The pellet was rinsed twice with 80% methanol by adding methanol and centrifuging for 15 min at 4000*g* each time. Following this, the methanol was gently decanted. Ultimately, pellets were lyophilized and preserved for subsequent analysis. In parallel, a protein-free substrate (PFS) prepared according to prior studies,^43^ consisting solely of fat and carbohydrates, was digested using the same protocol and analytical methods to quantify the contribution of digestive enzymes to both the background protein content and the overall mass and of the digests.

### 2.5 Quantification of Total Amino Groups by the O-Phthalaldehyde (OPA) Method

The total primary amine groups (R-NH_2_) in both the pellet (nonabsorbable fraction) and the supernatant (absorbable fraction) were quantified using the OPA assay according to^44^. Beforehand, the whole digesta pellet and 220 μL of supernatant—previously dried in a vacuum concentrator—were resuspended respectively in 4 and 1 mL of hydrolysis solution (6 M HCl, 0.0012% DPP, 24 mM NaOH, 0.4 nM norvaline) using hydrolysis-compatible glass vials. In parallel, 4 mg of each undigested sample was solubilized with 4 mL of the same hydrolysis solution. The vials were then flushed with N_2_ to prevent contact with oxygen,^45^ securely sealed, and placed in a 110 °C oven for 15–24 h. Next, the pellets and supernatant were filtered and diluted 10 and 5 times, respectively, with 0.1 M borate, and derivatized with an OPA solution (50 mM borate, 0.01% SDS, 0.8 g/L OPA in ethanol, 4 g/L Na-MES, 5 g/L Triton X-100). A glutamic acid—ranging from 0 to 8 mM in 0.1 M borate—was used as a standard curve.^46^ Finally, after a 10 min incubation at 30 °C in darkness, the obtained 1-alkylthio-2-alcylisonindol compounds were quantified by measuring absorbance at 340 nm using UV/vis spectrophotometry.

### 2.6 Determination of Total AA (TAA)

The TAA content of each product and its corresponding digest was measured following the AOAC 2018.06 standardized method.^47^ Briefly, after the acid hydrolysis described in 2.5, the samples were neutralized with 6 M NaOH, diluted 1/2 with 0.1 M HCl, filtered (0.45 μm, Branchia), and derivatized using the AccQ-Tag Ultra reagent in phosphate buffer.^48^ The resulting AA profiles were then analyzed by ultrahigh-performance liquid chromatography (UHPLC) coupled to a UV detector using an AccQ-Tag Ultra analytical column. Notably, under the applied hydrolysis conditions, tryptophan could not be quantified due to its degradation, while asparagine and glutamine were reported as their respective deamidated forms, aspartic acid and glutamic acid.

### 2.7 *In Vitro* Total Digestibility Calculation

The *in vitro* total digestibility of the products was evaluated using two distinct analytical methods. These comprised the quantification of total R-NH_2_ and AA in both the supernatant and pellet fractions, calculated according to the following equation, as previously described in^43^

1 invitrodigestibility(%)=((Fs‐Cs))/((Fs‐Cs)+max(0;Fp‐Cp))×100

where Fs = Meal supernatant, Cs = Protein-free control supernatant, Fp = Meal pellet, and Cp = Protein-free control pellet. The expression max­(0;Fp-Cp) ensures that the AA content from the protein-free blank digest is considered the minimum threshold, with any values falling below the enzyme background (due to analytical bias) being adjusted to zero.

### 2.8 Peptide Identification by Liquid Chromatography Coupled to Tandem Mass Spectrometry (HPLC-MS/MS)

The analyses were performed using a Vanquish Neo nano-UHPLC system connected to an Orbitrap Exploris 240 mass spectrometer. Before MS analysis, the dry digests were resuspended in 0.2% TCA and centrifuged at 21,000*g* for 5 min. Then, they were purified using ZipTipC18 pipet tips that were preconditioned with 50:50 acetonitrile/water containing 0.1% TFA, equilibrated with 0.2% TFA, and eluted in 50:50 acetonitrile/water +0.1% TFA. The eluates were dried under vacuum and reconstituted in 0.1% formic acid; 1 μL was injected for UHPLC–MS/MS analysis. The peptides were separated on an Easy-Spray Pepmap Neo C18 column using a gradient from 0 to 95% solvent B (80:20 acetonitrile/water +0.1% formic acid) over 64 min at 0.3 μL min^–1^, with solvent A consisting of water +0.1% formic acid. The Orbitrap Exploris 240 operated in positive ion mode, acquiring full MS scans over an *m*/*z* range of 350–1550 at 120,000 resolution, with RF lens at 70%, normalized AGC target of 300%, and an intensity threshold of 3.0 × 10^4^. MS/MS spectra were obtained at a resolution of 30,000 using a 2 Da isolation window, normalized AGC target of 50%, and normalized HCD collision energy of 30%. Spectra were analyzed using PEAKS Studio 11 (Bioinformatics Solutions Inc.) for de novo sequencing and database matching against a customized database containing the major soy proteins, including various genetic variants. Search parameters were set to no enzyme specificity, allowing up to two variable modifications per peptide: acetylation (N-terminal) and oxidation (Met) as variable modifications; precursor and fragment tolerances of 10 ppm and 0.02 Da, respectively. FDR threshold was set to 1% at the peptide-spectrum match (PSM) level using a target-decoy approach. Protein identification required a minimum 10LgP score of 15 and at least one unique peptide. Positive charge states +1 to +5 were allowed and peptide length was restricted to 5–45 amino acids. Peptide intensity normalization was performed using the default normalization method implemented in PEAKS Studio for label-free analysis, and only consistently detected peptides were retained for downstream analysis. The identified peptide sequences and their intensities were mapped onto the corresponding protein sequences using the Peptigram software (http://bioware.ucd.ie/peptigram) developed by^49^. This allowed for the comparison of peptide intensities and abundances across different regions of the proteins. The immunogenic potential of the identified peptides was evaluated through a predictive screening, performing sequence alignments against known immunogenic epitopes from soybean β-conglycinin subunits previously reported in chickens^50^ and in swine, bovine, and rats.^51^ Sequence similarity analyses and phylogenetic comparisons were conducted using the Molecular Evolutionary Genetics Analysis software (MEGA), applying a threshold of ≥90% sequence identity to detect relevant peptide matches.

### 2.9 Statistical Analysis

Results are expressed as mean ± standard deviation (SD) of three independent biological replicates (*n* = 3). Prior to hypothesis testing, the homogeneity of variances assumption was verified using the Brown-Forsythe test. Statistical comparisons were performed using one-way or two-way analysis of variance (ANOVA), followed by a Tukey-test for multiple comparisons. Specifically, one-way ANOVA was applied to evaluate protein digestibility measured by both the OPA and TAA methods, and two-way ANOVA was utilized to analyze individual amino acid digestibility, considering the enzymatic treatment and the specific amino acid as the main factors. Peptide abundance differences were evaluated based on sequence coverage rather than conventional ANOVA. Differences were considered statistically significant at *p* ≤ 0.05. All analyses were conducted using GraphPad Prism version 8.0.2 for Windows (GraphPad Software, San Diego, CA, USA).

## 3 Results and Discussion

### 3.1 *In Vitro* Digestibility

To evaluate overall protein and individual AA digestibility, both the supernatants and pellets obtained from methanol-precipitated intestinal digests were subjected to two distinct analytical methods after acid hydrolysis: (i) quantification of total R-NH_2_ using OPA and (ii) measurement of individual AA by UHPLC. In all cases, digestibility was calculated according to eq ([Disp-formula eq1]) detailed in Section 2.7. As shown in Table 1, the *in vitro* protein digestibility of untreated soybean meal was 83% and 89% when assessed by the OPA and TAA methods, respectively. These results are in good agreement with previously published *in vivo* data in pigs, where protein digestibility for soybean meal typically ranges from 80 to 88%.^52,53^ Proteolytic treatment with Protease I led to a statistically significant improvement in digestibility (*P* < 0.05) as determined by OPA and TAA, 90% and 96%, respectively, indicating a robust enhancement in protein hydrolysis efficiency. In contrast, treatment with Protease II also improved digestibility, reaching 91%, but this value was only significantly different from the Control when measured by the OPA method. This discrepancy can be explained by the fact that the OPA assay quantifies N-primary amines but does not effectively detect those associated with proline residues.^54^ Consequently, this limitation can lead to an underestimation of the OPA-based digestibility values in the Control sample, where the presence of proline in the generated peptides is slightly higher at the N-terminal position. Similarly, an increased individual AA digestibility was observed with Protease I and to a lesser extent with Protease II (Supporting Information Table S1). Figure 1 shows the *in vitro* digestibility of indispensable AA. Control soybean meal digestible indispensable AA values were consistent with previously reported *in vivo* data.^52,53,52,53^ An enhanced AA digestion was observed upon enzymatic treatment, particularly with Protease I. The increase in the individual AA digestibility agrees with the overall improvement observed in protein digestibility and highlights the potential nutritional benefits of enzymatic supplementation.

**Figure 1:**
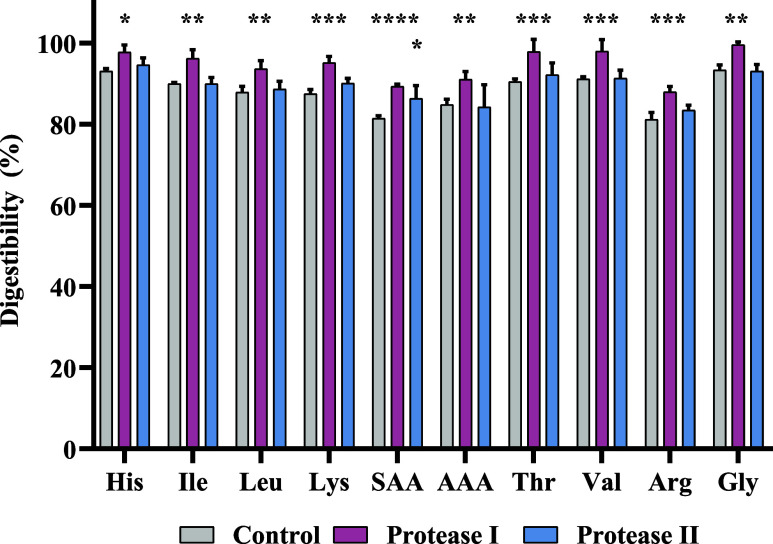
*In vitro* digestibility of indispensable amino acids (IAA). Percentage values of i*n vitro* digestibility for each essential amino acid are presented for untreated (Control) and treated soybean meal (Protease I and Protease II), based on the quantification of each amino acid in the absorbable and nonabsorbable fractions employing ultrahigh-performance liquid chromatography (UHPLC) integrated with a UV detector. Data are expressed as the mean ± standard deviation (SD) from three independent experiments (*N* = 3). Asterisks denote statistically significant differences relative to the Control for each amino acid: *p ≤ 0.05; **, *p* ≤ 0.01; ***, *p* ≤ 0.001; ****, *p* ≤ 0.0001.

**Table 1:** *In Vitro* Protein Digestibility^a^

	protein digestibility (%)	
sample code	TAA	OPA
Control	88.9 ± 1.0^b^	83.3 ± 0.4^b^
Protease I	95.5 ± 1.8^a^	89.7 ± 1.4^a^
Protease II	91.5 ± 0.7^ab^	91.2 ± 2.4^a^

a *In vitro* protein digestibility values (expressed as percentages) for untreated (Control) and protease-treated soybean meal (Protease I and Protease II) are shown. Digestibility was assessed by quantifying total amino acids (TAA) in the absorbable and nonabsorbable fractions using ultrahigh-performance liquid chromatography (UHPLC) with UV detection, as well as total amino groups (R-NH_2_) using the o-phthalaldehyde (OPA) assay. All data represent the mean ± standard deviation (SD) obtained from three independent experiments (*N* = 3). Different superscript letters within the same column indicate statistically significant differences in average digestibility among the different samples.

In addition, Supporting Figure S1 shows that the original soybean meal sample displays the characteristic SDS-PAGE bands of the major storage proteins β-conglycinin and glycinin. Consistent with previous reports, the subunits of β-conglycinin (α, α′, β) migrate at approximately 72 kDa, 76 kDa, and 52 kDa, respectively,^55^ while glycinin typically shows acidic A-chain bands of ∼32–40 kDa and basic B-chain bands of ∼18–20 kDa.^16,56^ In contrast, these storage-protein bands are no longer detectable in the soluble (absorbable) fractions obtained after *in vitro* digestion, which instead display electrophoretic bands under 15 kDa, corresponding to degradation products, and bands between 20 and 50 kDa that match those observed in the protein-free substrate controls, confirming that these bands correspond to the digestive enzymes present in the system—such as pancreatic α-amylase, with a molecular weight of ∼53 kDa,^57^ and other pancreatic proteases falling within the ∼24–29 kDa range^58^—which match those observed in the protein-free substrates, confirming that the soluble fraction contains only enzyme-derived proteins and no intact soybean storage proteins. These findings align with previous studies,^59^ which reported similar enzyme-related band patterns after digestion of pea flour, with proteolytic fragments appearing as lower molecular weight bands over time.

### 3.2 Peptide Analysis by LC-MS/MS

#### 3.2.1 Protein Sequence Coverage

Mass spectrometric analysis and subsequent data processing allow the identification of the peptide fingerprint after simulated gastrointestinal digestion of each diet. It is important to consider that LC-MS/MS detection depends heavily on peptide ionization efficiency and that, under the applied analytical conditions, detection is limited to peptides containing at least five AA, while the upper limit is defined by their solubility in 0.2% TCA. As illustrated in Figure 2, the sequence coverage of major soybean proteins varied depending on the protease added before *in vitro* digestion. Samples treated with Protease I and II generally showed higher sequence coverage across most target proteins compared to the Control sample, indicating the hydrolysis, by the joint action of the added proteases and the gastrointestinal enzymes, of long fragments that precipitate in the Control sample. However, the extent of this improvement varied with the enzyme employed and the protein. Protease II appeared to yield a higher number of detectable fragments for the subunits of β-conglycinin, whereas Protease I exhibited slightly higher effectiveness in releasing detectable peptides from the glycinin subunits. In particular, Protease II demonstrated greater efficacy, particularly for the β-conglycinin β-subunit, Glycinin G1, and β-conglycinin α-subunit, with coverage values of 76.92%, 73.74%, and 66.38%, respectively. Conversely, proteins such as Glycinin G5 showed higher coverage with Protease I (52.64%) compared to Protease II (42.48%).

**Figure 2:**
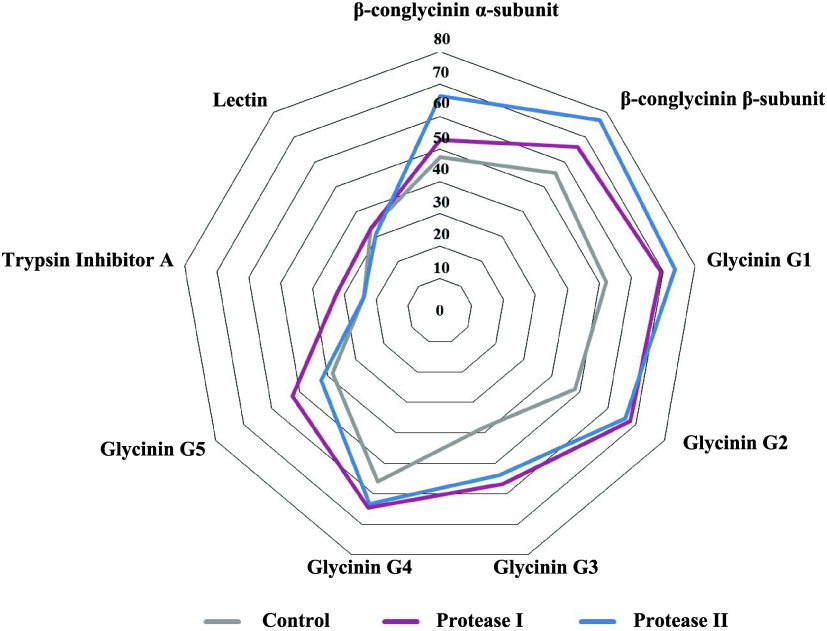
Protein sequence coverage in *in vitro* digests of soybean meal under different treatments for specific targets. Sequence coverage (in %) of β-conglycinin α-subunit, β-conglycinin β-subunit, Glycinin (G1-G5), Trypsin inhibitor A, and Lectin based on peptides identified in *in vitro* digests of soybean meal samples without treatment (Control) and those treated with Protease I and Protease II. All peptide sequences were obtained through LC-MS/MS analysis using a 240 Orbitrap mass spectrometer (Thermo Fisher Scientific, San Jose, CA, USA), coupled with a nano ultrahigh-performance liquid chromatography (UHPLC) Vanquish Neo (Thermo Fisher Scientific, USA), and mapped to the corresponding protein sequences.

Protease treatments also led to modest improvements in the number of identified peptides from antinutritional proteins, such as Trypsin inhibitor A and Lectin, with Protease I exhibiting a slightly higher effectiveness in targeting these proteins. These observations highlight the clear differences between the two proteases, suggesting that the joint action of multiple enzymes in a formulation has a greater potential for overhydrolysis compared to more specific, single-protease approaches.

Interestingly, this diversification of the peptide profile was not accompanied by a significant increase in the release of free AA, as shown in Supporting Information Figure S2. No significant differences were observed in the total quantification of free AA between the Control and both protease treatments. The findings indicate that Proteases I and II mainly act as endopeptidases, since they break down major soybean proteins into a broad range of peptide sizes, without causing a notable rise in free AA levels when compared with the Control group. Hydrolyzing soybean proteins into smaller peptides can improve nutrition, as smaller peptides are absorbed faster from the small intestine than free AA in chickens.^60^

Overall, the results indicate that the addition of proteases enhances the digestibility of soybean proteins, consistent with previous studies demonstrating that exogenous protease supplementation improves protein hydrolysis and AA availability in plant-based feed ingredients.^28,29^

#### 3.2.2 Peptide Distribution and Uniqueness across Diets

To visualize the distribution of peptides generated after simulated digestion, the heatmap in Figure 3 represents the positional frequency of AA occurrences within the identified peptides, aligned along the sequences of the proteins (A) β-conglycinin α-subunit, (B) Glycinin G1, and (C) Trypsin inhibitor A under the three experimental conditions: Control, Protease I, and Protease II. This representation provides qualitative insights into protein coverage and highlights regions where peptides were identified, although it does not convey quantitative data or relative abundances. Green, yellow, and red tones indicate low, intermediate, and high peptide representation, respectively.

**Figure 3:**
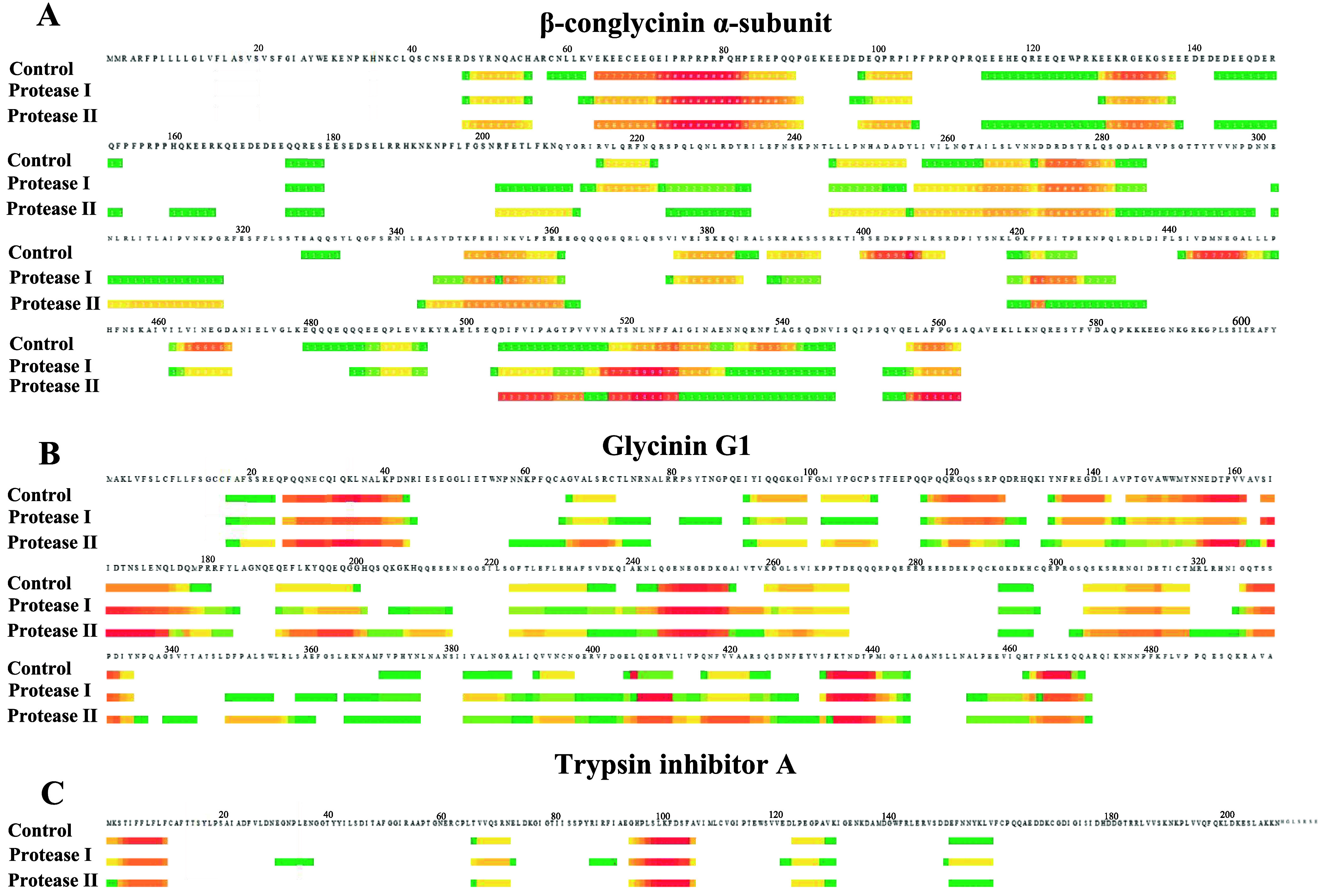
Comparative heatmap profiles of selected soybean proteins following *in vitro* digestion with or without protease treatment. Alignment of peptides identified in (A) β-conglycinin α-subunit, (B) Glycinin G1, and (C) Trypsin inhibitor A showing the distribution and intensity of peptides identified in *in vitro* digests of soybean meal samples under different conditions: untreated (Control) and treated with Protease I or Protease II. Peptides were identified through LC-MS/MS analysis using a 240 Orbitrap mass spectrometer (Thermo Fisher Scientific, San Jose, CA, USA), coupled with a nano ultrahigh-performance liquid chromatography (UHPLC) Vanquish Neo (Thermo Fisher Scientific, USA), and mapped to their respective protein sequences.

In the Control condition of β-conglycinin α-subunit (Figure 3A), a clear clustering of peptides with high frequency (red color) is observed, particularly in the region 64–83. By the use of exogenous proteases, the frequency of appearance of some regions increased, *e.g*., at the carboxy tail region (from residue 500 onward). In addition, some peptides from the β-conglycinin α-subunit not identified in the Control condition were identified with the use of proteases. This is the case of ^202^SPQLQNLRDY^211^, ^223^LLPNHADADYL^234^, and ^302^LSSTEAQQSYLQGFSR^317^. On the other hand, certain peptides were detected in the Control sample but were absent in the samples treated with Protease I and II: ^58^PQHPE^62^, ^328^FEEIN^332^, ^400^FFEITPEKNPQ^410^, and ^441^LVINEGDANIELV^453^. This may be attributed to the action of the added proteases, which likely hydrolyze these peptide fragments, generating peptides shorter than five AA that fell below the identification threshold under the analytical conditions employed.

These patterns are complemented by the Venn diagram (Figure 4), which shows the unique and shared peptide sequences identified across the three samples for a given protein. For β-conglycinin α-subunit, some peptides, such as ^349^LQESVIVE^356^ and ^376^TISSEDKPFNL^386^, were found exclusively in the Control sample, but these peptides were further hydrolyzed to shorter forms, ^349^LQESVIV^355^ and ^376^TISSEDKPF^384^, by the addition of Protease I (Figure 4A). The other pattern observed in this protein corresponds to peptide regions that were not detected in the Control sample but were identified in one or both protease-treated samples. Representative examples of these fragments include ^202^SPQLQNLRDY^211^, ^223^LLPNHADADYL^234^, ^302^LSSTEAQQSYLQGFSR^317^, and ^428^LLLPH^432^, which are part of the 17 peptides from the β-conglycinin α-subunit commonly identified between the protease-treated samples, as shown in the Venn diagram (Figure 4A). Their absence in the Control sample could be attributed to these sequences being embedded in long, high-molecular-weight peptides that likely precipitated during the cleanup step involving 0.2% TCA. The protein Glycinin G1 displays a coverage pattern markedly affected by the protease treatment, as observed in both the heatmap and the Venn diagram (Figures 3B and 4B, respectively). The heatmap shows a broader and more continuous distribution of detected fragments in the protease-treated samples as compared to the Control sample, i. e. regions 206–214, 348–369, 398–404, and 454–461. This observation is further supported by the Venn diagram (Figure 4B), where 66 and 36 peptides were exclusively identified in the Protease I and Protease II conditions, respectively. A core set of 83 peptides was shared across all three conditions, suggesting the presence of stable or commonly detectable regions regardless of enzymatic treatment. Similarly, a group of 32 peptides shared between Protease I and Protease II (but not present in the Control) suggests the presence of regions that become accessible only after protease addition, reinforcing the role of an assisted proteolysis in uncovering the peptide architecture of this protein.

**Figure 4:**
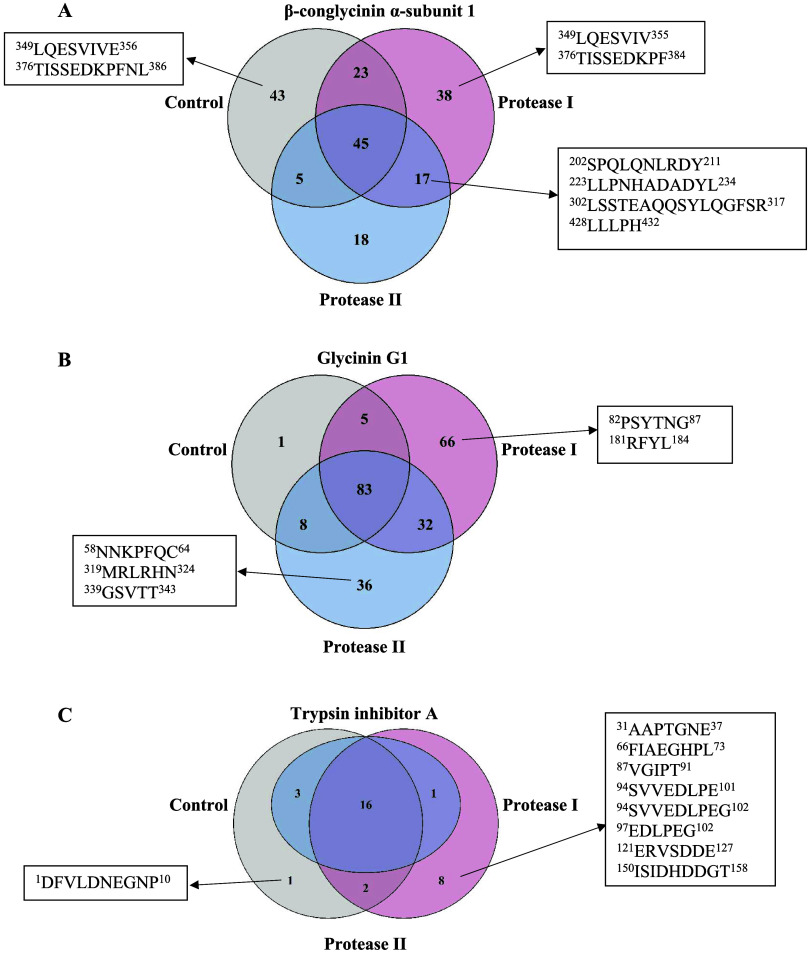
Venn diagrams showing peptide distribution among samples for selected protein targets. Unique and shared peptides identified in untreated (Control) and treated soybean meal (Protease I and Protease II) samples for (A) β-conglycinin α-subunit, (B) β Glycinin G1, and (C) Trypsin inhibitor A are shown. For each protein target, some example peptide sequences from each sample are presented. Peptides were identified by LC-MS/MS analysis using a 240 Orbitrap mass spectrometer (Thermo Fisher Scientific, San Jose, CA, USA), integrated with a nano ultrahigh-performance liquid chromatography (UHPLC) Vanquish Neo (Thermo Fisher Scientific, USA).

In contrast to β-conglycinin α-subunit and Glycinin G1, the peptide coverage pattern for Trypsin inhibitor A (Figure 2) was comparatively more limited, both in terms of the number and distribution of detected peptides. This is reflected in the heatmap (Figure 3C), which shows only a few colored regions, mostly with low or moderate intensity signals (green/yellow), and large areas entirely lacking signal throughout the protein sequence. This sparse coverage reflects the low susceptibility to proteolysis. The peptides from Trypsin inhibitor A found to be unique to Protease I are shown in Figure 4C and no unique fragment was found for Protease II. This suggests that Trypsin inhibitor A is resistant to hydrolysis to gastrointestinal enzymes and to both proteases tested, although slightly more susceptible to Protease I, underscoring that a single protease is insufficient and highlighting the need for a multienzyme approach for efficient digestion. This resistance may be attributed to the compact tertiary structure and multiple disulfide bonds that stabilize the conformation of trypsin inhibitors, as previously described.^61^ The rigid architecture limits enzymatic accessibility, thereby hindering both peptide generation and subsequent detection during proteomic analysis, since samples were not reduced and alkylated prior analysis.

#### 3.2.3 Cleavage Site Specificity and Protease Action

Regarding proteolytic cleavage patterns, Figure 5 shows the absolute frequency of AAs observed at the amino-terminal (Nt) and carboxy-terminal (Ct) ends of peptides derived from *in vitro* digests of untreated (Control) and treated soybean meal with Protease I and Protease II. At the Nt end, serine was the predominant AA in both the Control and Protease II-treated samples, whereas leucine was the most frequent residue in peptides resulting from Protease I treatment. Conversely, at the Ct, proline was the prevalent common AA in both the Control and Protease II samples, glutamine and glutamic acid dominated in the Protease I sample, followed by proline. In the Control, the observed cleavage sites are due to the action of the gastrointestinal enzymes present in the *in vitro* digestion. The presence of proline at the Ct may reflect steric hindrance to cleavage by enzymes like chymotrypsin, which typically targets hydrophobic residues but is inefficient when proline immediately follows the cleavage site.^62^ In addition, the persistence of proline residues may be attributed to their location within proline-rich motifs commonly found in plant proteins. For example, repetitive sequences like Pro–Pro–Val–Tyr–Lys identified in soybean cell wall proteins have been associated with enhanced structural stability and resistance to proteolysis.^63^ This is further supported by the known conformational role of proline, which disrupts canonical secondary structures and promotes the formation of rigid polyproline helices that can limit protease accessibility.^64^ Protease I appears to shift the cleavage pattern, favoring Leu at the Nt and Gln and Glu at the Ct. This distinct profile highlights the synergistic action of the multiprotease blend, which combines acidic, neutral, and alkaline enzymes from *A. niger*, *B. subtilis*, and *B. licheniformis*, and explains why Protease I achieved a superior performance in enhancing overall digestibility. For instance, the prominence of Nt-Leu aligns directly with the substrate specificity of *B. subtilis* neutral proteinases, which preferentially cleave peptide bonds where the amino acid donating the amino group is a hydrophobic residue, with leucine facilitating the highest hydrolysis rate.^65,66^ Furthermore, the *A. niger* acidic proteases specifically target bulky or aromatic residues on both sides of the cleavage site (such as Leu, Phe, or Tyr).^67^ In contrast, the use of Protease II, a single alkaline protease derived solely from *B. licheniformis*, caused apparently a higher frequency at the Nt of Ser, and Arg and Lys at Ct, although it has been reported in the literature that alkaline proteases typically exhibit a broad specificity with no apparent regularity when acting on large molecular substrates.^68^

**Figure 5:**
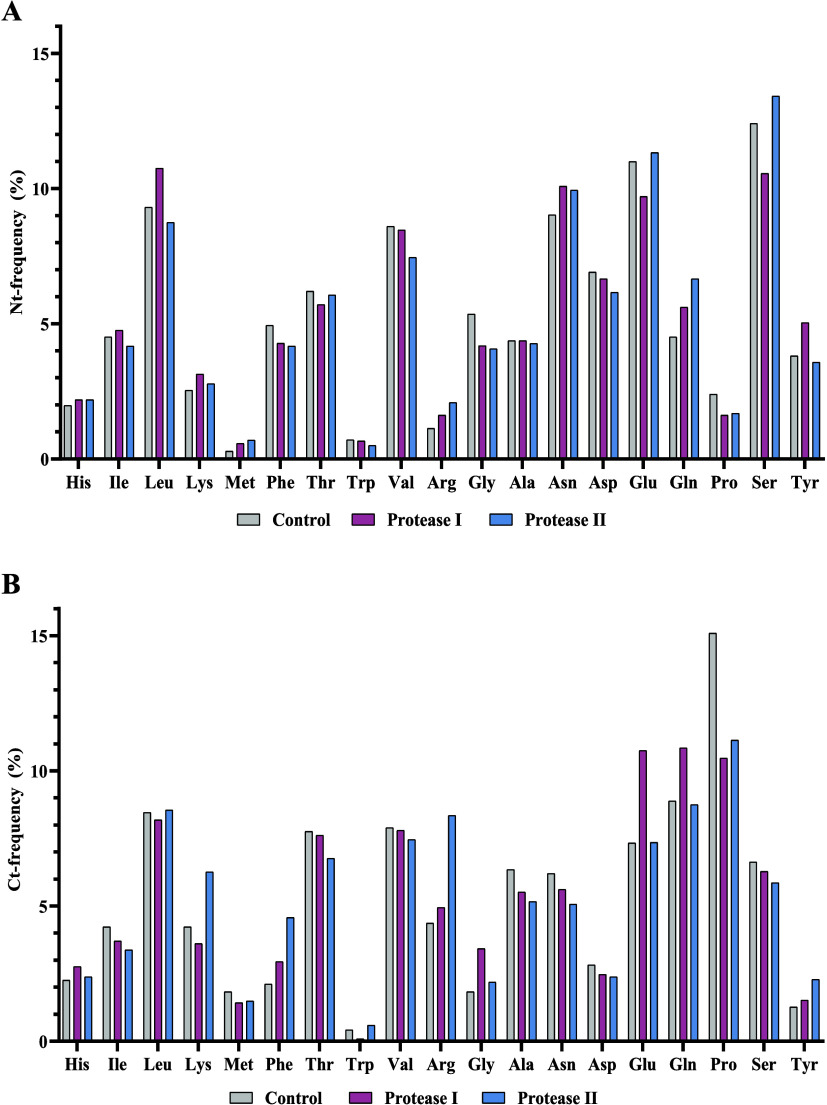
Cleavage site frequency at peptide termini across samples. Relative frequency (in %) of cleavage events occurring at each amino acid residue at the amino-terminal (Nt) (A) and carboxy-terminal (Ct) (B) positions in peptides derived from nontreated (Control) and protease-treated (Protease I and Protease II) soybean meal digests. Peptides were identified by LC-MS/MS using a 240 Orbitrap mass spectrometer (Thermo Fisher Scientific, San Jose, CA, USA) coupled with coupled with a nano ultrahigh-performance liquid chromatography (UHPLC) Vanquish Neo (Thermo Fisher Scientific, USA).

Altogether, these features demonstrate that the addition of exogenous proteases introduces a broader and more diverse proteolytic profile, enhancing hydrolysis even in structurally resistant domains. In addition, these findings align with the different AA compositions of the digestion-resistant peptides presented in Supporting Information Figure S3. As anticipated, protease-containing soybean meal digests exhibited lower relative abundances of Pro and Asp compared with the Control, providing insights into protease-specific substrate preferences and potential resistance structural hotspots within soybean proteins.

The results demonstrate that supplementation with exogenous proteases significantly enhances protein hydrolysis beyond the level achieved by the endogenous digestive enzymes alone. The increased sequence coverage observed suggests improved cleavage of resistant or structured protein domains, likely because of the specific substrate recognition and cleavage preferences of the protease used.

#### 3.2.4 Identification and Homology of Soybean-Derived Peptides with Known Immunogenic Epitopes in Different Animal Species

To evaluate the potential immunogenicity of soybean-derived peptides following gastrointestinal digestion, we conducted a comparative analysis between peptides identified under the three experimental conditions (Control, Protease I and Protease II) and previously reported immunogenic epitopes from chickens^50^ and from swine, bovine, and rats.^51^ It is important to note that this assessment is based only on *in silico* epitope sequence comparisons and peptide fragmentation profiling. The comparative results are summarized in Table 2, which lists the previously reported immunogenic epitopes and their corresponding peptide matches in each condition, along with the associated protein subunits with accession numbers. Shared regions are indicated in bold, and peptides longer than eight AA, commonly associated with immunogenicity,^69^ are specifically underlined to highlight their relevance. Most of these peptides showing homology to known epitopes originated from soybean glycinin G1 and β-conglycinin, two major allergens in soybean.^17,18^ To ensure accuracy, the search focused exclusively on linear epitopes with a minimum of 90% sequence identity. Across the conditions, several peptides exhibited partial or complete sequence overlap with known epitopes, showing distinct detection patterns depending on the condition.For example, the epitope TLFENQNGRIRLLQRFNKRSP was represented by the matching peptide fragment SSNSFQ **TLFENQNGR** in the Control, but it was completely absent in the protease-treated samples, suggesting that enzymatic hydrolysis may have degraded this immunogenic region. This observation is consistent with previous reports indicating that proteolytic digestion can reduce exposure to certain allergenic peptides and might theoretically modulate subsequent immune activation.^27^ For other epitopes, both Control and protease-treated samples showed matching immunogenic fragments, suggesting that these sequences are relatively resistant to enzymatic cleavage and may represent stable immunogenic motifs within the soybean protein structure. However, despite the presence of these conserved regions, a progressive fragmentation of the immunogenic sequences was also observed under enzymatic hydrolysis, which could potentially alter their exposure or recognition by the immune system. For example, in epitopes such as LQESVIVEISK and EDENNPFYFR, shorter nonimmunogenic fragments (*e.g*., VIVEIS, EDENNPF) were generated in the treated samples, indicating partial degradation of the immunogenic core regions. Conversely, epitopes such as PHFNSKAIVILVINEGD and LLLPNHADADYLIVILNGT were not detected in the Control but identified in both Protease I and Protease II conditions (*e.g*., **LLPNHADADYL**). This pattern could be attributed to the presence of long, insoluble protein fragments comprising these epitopes in the untreated sample—likely precipitated during the cleanup step involving 0.2% TCA—which are not detectable by mass spectrometry, rather than to the absence of immunogenic sequences in the Control conditions. This interpretation is consistent with previous findings, including those reported by^50^, who identified these epitopes in untreated soybean β-conglycinin, suggesting that the sequences are indeed present but not accessible under the analytical conditions used. Therefore, the protease treatment likely resulted in key cleavages thereby facilitating the detection of epitope-containing peptides that remained undetectable in the Control.

**Table 2:** Identification of Soybean Peptides and Their Associated Immunogenic Epitopes in Different Animal Species^a^,^b^,^c^

epitope	accesion number	Control	Protease I	Protease II
TLFENQNGRIRLLQRFNKRSP^c^	F7J077	SSNSFQ **TLFENQNGR**	**-**	**-**
VPSGTTYYVVNPDNNENLR^c^	P0DO16/P11827	**VVNPDNNENLR** LI	**VVNPDNNENLR** LI	**VVNPDNNENLR**
		**YVVNPDNNEN**	**YVVNPDNNEN**	**YVVNPDNNENLR**
		**VVNPDNNEN**	**YVVNPDN**	**VVNPDNDEN**
		**VPSGTT**	**YVVNPD**	**VPSGTT**
			**VPSGTT**	
LQESVIVEISK^c^	P0DO16/P11827	**LQESVIVEISK** EQ	**LQESVIVEISK** EQ	**LQESVIVEISK** EQ
		**LQESVIVEISK** E	**LQESVIVEISK** E	**LQESVIVEISK** E
			**LQESVIVEISK**	**LQESVIVEISK**
			**VIVEIS**	**VIVEIS**
EDENNPFYFR^c^	F7J077/P25974	**EDENNPFY** LR	**EDENNPFY** LR	**EDENNPFY** LR
		**EDENNPFY** L	**EDENNPFY** L	**EDENNPF**
			**EDENNPF**	
AILTLVNNDDR^c^	P0DO16/P11827/F7J077	**TLVNNDDR** DS	**TLVNNDDR** DS	**TLVNNDDR** DS
		**LVNNDDR** DS	**LVNNDDR** DS	**SLVNNDDR** DS
				**TLVNN**
DLDIFLSSVDINEGALLLPHFNSK^c^	P0DO16/P11827/F7J077	**SVDINEGALLLPH**	**SSVDINEGALLLPH**	**SSVDINEGALLLPHFNSK**
		**DINEGALLLPH**	**SVDINEGALLLPH**	**SVDINEGALLLPH**
		**SSVDINEGA**	**DINEGALLLPH**	**DINEGALLLPHFNSK**
		**SVDINEGA**	**SSVDINEGA**	**DINEGALLLPH**
		**VDINEGA**	**SVDINEGA**	**SSVDINEGA**
			**VDINEGA**	**SVDINEGA**
			**DINEGA**	**DINEGA**
			**FNSKPN**	
SRDPIYSNKLGKFF^c^	P0DO16/P11827	**SRDPIYS**	**SRDPIYS**	**SRDPIYSNKLGK**
		**SRDPI**	**DPIYS**	**SRDPIYSNKL**
		**DPIYS**		**SRDPIYS**
				**SRDPI**
				**DPIYS**
PHFNSKAIVILVINEGD^c^	P04776		**EGD**LIAVPT	**EGD**LIAVPT
			**EGD**LIA	**EGD**LIA
			**GD**LIAVP	**GD**LIAVP
LLLPNHADADYLIVILNGT^c^	P0DO16		**LLPNHADADYL**	**LLPNHADADYL**
			**LLPNHADAD**	

a Peptides identified from soybean proteins and previously reported related epitopes in chicken^50^ and in swine, bovine, and rats^51^ are shown for untreated (Control) and protease-treated soybean meal (Protease I and Protease II). Matching sequences are listed along with their corresponding accession numbers. Shared regions among peptides are indicated in bold, and sequences longer than eight amino acids are underlined. Accession numbers correspond to the following soybean proteins: F7J077 (Glycinin G1 subunit), P0DO16 (Glycinin G2 subunit), P11827 (β-conglycinin α’-subunit), P25974 (Glycinin G4 subunit), and P04776 (β-conglycinin α-subunit).

b Epitope obtained from chicken.^50^

c Epitope reported in swine, bovine, and rats.^51^

Overall, these findings highlight the differential impact of protease treatment on protein and amino acid digestibility and the exposure, degradation, or fragmentation of immunogenic regions. These data describe variations in potential peptide exposure rather than changes in biological immunogenicity. Consequently, target validation through specific *in vitro* immunological assays or *in vivo* challenges should be used to confirm immunological reduction. Moreover, evaluating amino acid bioavailability and feed conversion efficiency in chickens is required to determine the nutritional impact of exogenous proteases, together with derived gut health implications. Finally, it remains to be established that the economic relevance of protease supplementation.

Therefore, taken together, these results emphasize the broad potential of enzymatic treatments to improve digestibility and potentially reduce the allergenic risk. Further research incorporating *in vitro* and *in vivo* validation will be essential to validate these findings and fully assess the implications for improving nutrient utilization and safety across both human and animal nutrition.

## Supplementary Material



## Notes

*In vitro* amino acid and protein digestibility (Table S1); electrophoretic profile of soybean meal and absorbable digests (Figure S1); free amino acid quantification in the digests (Figure S2); amino acid composition of digestion-resistant peptide fragments in *in vitro* digests of untreated and protease-treated soybean meal samples (Figure S3) (PDF)

## Abbreviations

AA: amino acid
ANFs: antinutritional factors
TFA: trifluoroacetic acid
TCA: trichloroacetic acid
SDS-PAGE: sulfate–polyacrylamide gel electrophoresis
SSF: simulated salivary fluid
SGF: simulated gastric fluid
SIF: simulated intestinal fluid
PFS: protein-free substrate
OPA: o-phthalaldehyde
R-NH2: primary amine groups
TAA: total amino acid
UHPLC: ultrahigh-performance liquid chromatography
HPLC-MS/MS: high-performance liquid chromatography coupled to tandem mass spectrometry
MEGA: molecular evolutionary genetics analysis software
SD: standard deviation
ANOVA: analysis of variance
Nt: amino-terminal
Ct: carboxy-terminal
