# Supplementary material for: Improving In Vitro Digestibility and Reducing Immunogenic Peptide Exposure in Soybean Meal through Exogenous Protease Supplementation
Source: J Agric Food Chem. 2026 Jun 29;74(27):21673–84. doi: 10.1021/acs.jafc.6c05174 (PMC13383720; doi:10.1021/acs.jafc.6c05174)
Supplement: Supplementary file 1 [file jf6c05174_si_001.pdf]

Improving *In Vitro* Digestibility and Reducing Immunogenic Peptide Exposure in Soybean Meal through Exogenous Protease Supplementation

Leila Zafra[1,2], Cristina Jiménez-Holgado[1], Bindhu Lakshmibai Vasanthakumari[3], Beatriz Miralles[1], Marta Martinez-Sanz[1], Isidra Recio[1]*

[1]Institute of Food Science Research, CIAL (CSIC-UAM, CEI UAM+CSIC), C. Nicolás Cabrera, 9, 28049 Madrid, Spain

[2]Escuela de Doctorado, Universidad Autónoma de Madrid (UAM), C. Francisco Tomas y Valiente, 7, 28049, Madrid, Spain

[3]Kemin Industries, 1900 Scott Ave, Des Moines, IA 50317, Iowa, USA

*Corresponding author: E-mail address: i.recio@csic.es

Phone: +34 910017940

Fax: +34 910017905

Nicolás Cabrera, 9. 28049 Madrid, Spain

Table S1. ***In vitro* amino acid and protein digestibility**. Individual amino acid and protein *in vitro* digestibility values (in %) are shown for untreated (Control) and protease-treated soybean meal (Protease I and Protease II), based on quantification in the absorbable and non-absorbable fractions of total amino acids (TAA) using ultra-high performance liquid chromatography (UHPLC) in combination with UV detection. Data are expressed as mean ± standard deviation (SD) from three independent experiments (N = 3). Different superscript letters within the same row denote statistically significant differences in average digestibility among the samples.

	**Amino Acid Digestibility (%)**		
**Sample code**	**Control**	**Protease I**	**Protease II**
**His**	93.0 ± 0.7^{b}	96.7 ± 2.2^{a}	93.6 ± 2.2^{ab}
**Ile**	89.9 ± 0.4^{b}	93.9 ± 4.2^{a}	88.1 ± 3.4^{b}
**Leu**	87.8 ± 1.5^{b}	91.6 ± 3.7^{a}	86.3 ± 4.3^{b}
**Lys**	87.5 ± 1.1^{b}	93.2 ± 3.5^{a}	88.7 ± 2.5^{b}
**SAA**	81.4 ± 0.6^{b}	86.9 ± 4.1^{a}	85.2 ± 3.0^{a}
**AAA**	84.8 ± 1.4^{b}	88.7 ± 4.2^{a}	84.2 ± 3.9^{b}
**Thr**	90.4 ± 0.7^{b}	95.6 ± 4.5^{a}	90.3 ± 3.8^{b}
**Val**	91.1 ± 0.5^{b}	95.7 ± 4.4^{a}	89.3 ± 3.6^{b}
**Arg**	81.2 ± 1.7^{b}	85.4 ± 4.5^{a}	82.2 ± 2.3^{b}
**Gly**	93.3 ± 1.3^{b}	98.1 ± 2.5^{a}	92.2 ± 1.9^{b}
**Ala**	91.6 ± 1.0^{b}	97.3 ± 2.4^{a}	91.3 ± 3.4^{b}
**Asp**	92.2 ± 0.8^{b}	99.6 ± 0.6^{a}	94.3 ± 1.6^{b}
**Glu**	88.5 ± 1.2^{b}	93.5 ± 1.1^{a}	89.2 ± 1.4^{ab}
**Pro**	87.0 ± 2.1^{b}	95.5 ± 3.5^{a}	87.5 ± 2.6^{b}

**Ser**	88.8 ± 1.4^{b}	96.3 ± 3.1^{a}	89.6 ± 3.6^{b}

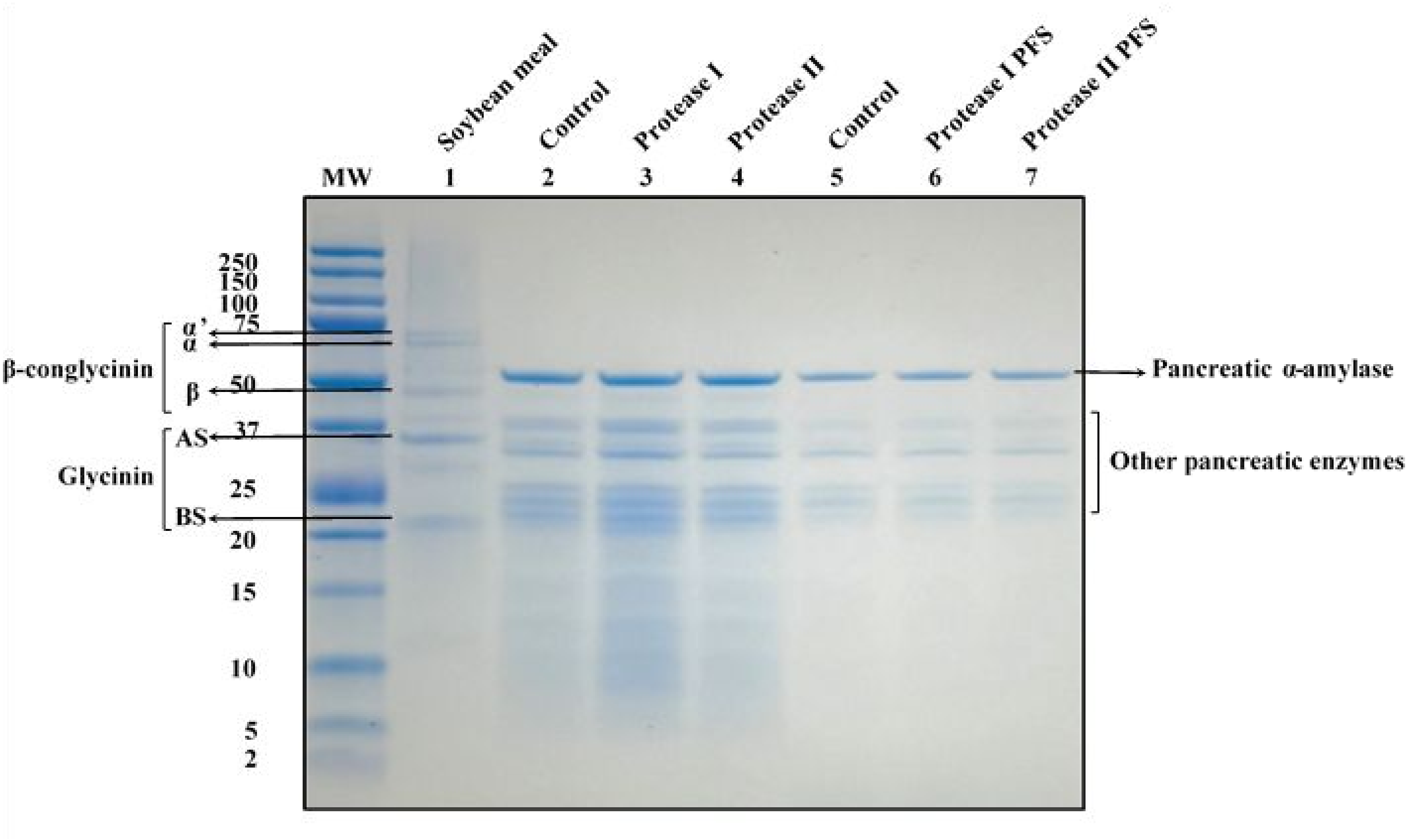

Figure S1. **Electrophoretic profile of soybean meal and absorbable digests**. SDS-PAGE at 12% (12% Criterion™ XT Bis-Tris Protein Gel, Bio-Rad, Richmond, CA, USA) of soybean meal and six supernatants corresponding to the soluble (absorbable) fraction from the *in vitro* digestion of untreated (Control) and protease-treated soybean meal (Protease I and Protease II) and their corresponding protein-free product (Control PFS, Protease I PFS and Protease II PFS) serving as the enzyme control. All the samples were dissolved in loading buffer (0.05 M Tris-HCl pH 6.8; 1.6 % w/v SDS; 8 % v/v glycerol; 0.002 % w/v bromophenol blue; 2 % v/v β-mercaptoethanol) at a concentration of 3 mg/mL. The loaded sample amount was 120 µg. The molecular weight marker (MW) used was Precision Plus Dual Xtra Prestained Protein Standard from Bio-Rad.

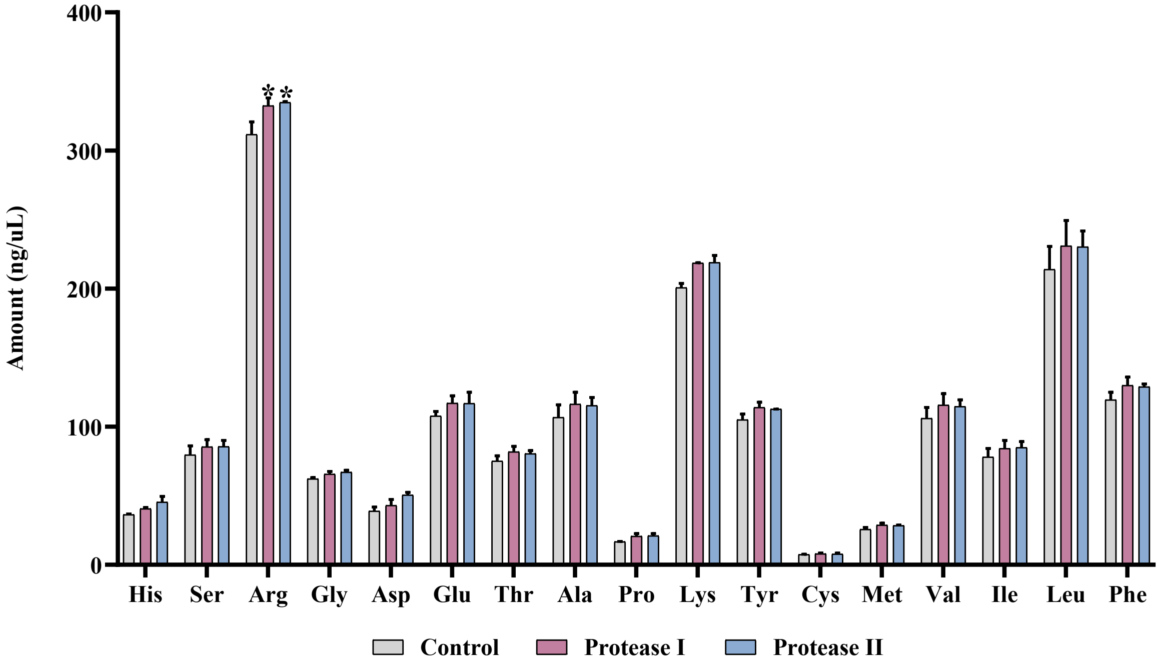

Figure S2. **Free amino acid quantification in the digests**. The amount (in ng/µL) of free amino acids measured in the obtained soloubIe fraction resulting from the *in vitro* digestion of untreated (Control) and protease-treated soybean meal (Protease I and Protease II) is shown. Quantification was carried out using ultra-high performance liquid chromatography (UHPLC) in combination with UV detection. Asterisks denote statistically significant differences relative to the Control for each amino acid: $p \leq 0.05$.

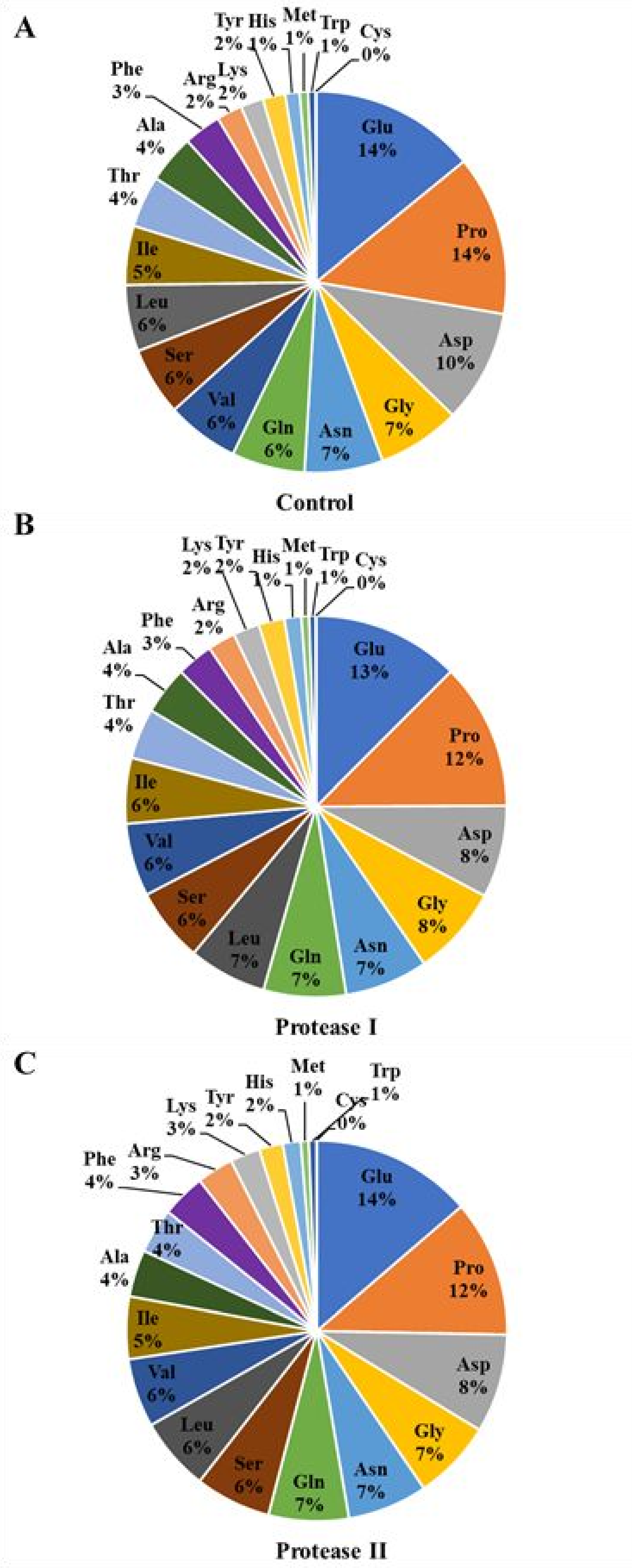

Figure S3. **Amino acid composition of digestion-resistant peptide fragments in *in vitro***

digests of untreated and protease-treated soybean meal samples. Pie charts showing the relative abundance (in %) of each amino acid present in peptides resistant to *in vitro* digestion of soybean meal under different treatments: Control untreated **(A)**, treated with Protease I **(B)**, and treated with Protease II **(C)**. The amino acid profiles reflect the composition of peptides remaining after *in vitro* simulated gastrointestinal digestion, as identified by LC-MS/MS using a 240 Orbitrap mass spectrometer (Thermo Fisher Scientific™, San Jose, CA, USA), coupled with a nano ultra-high performance liquid chromatography (UHPLC) Vanquish Neo system (Thermo Fisher Scientific™, USA).
